# Landscape Context Mediates Avian Habitat Choice in Tropical Forest Restoration

**DOI:** 10.1371/journal.pone.0090573

**Published:** 2014-03-04

**Authors:** J. Leighton Reid, Chase D. Mendenhall, J. Abel Rosales, Rakan A. Zahawi, Karen D. Holl

**Affiliations:** 1 Department of Environmental Studies, University of California Santa Cruz, Santa Cruz, California, United States of America; 2 Center for Conservation Biology, Department of Biology, Stanford University, Stanford, California, United States of America; 3 Organization for Tropical Studies, San Vito de Coto Brus, Costa Rica; University of Marburg, Germany

## Abstract

Birds both promote and prosper from forest restoration. The ecosystem functions birds perform can increase the pace of forest regeneration and, correspondingly, increase the available habitat for birds and other forest-dependent species. The aim of this study was to learn how tropical forest restoration treatments interact with landscape tree cover to affect the structure and composition of a diverse bird assemblage. We sampled bird communities over two years in 13 restoration sites and two old-growth forests in southern Costa Rica. Restoration sites were established on degraded farmlands in a variety of landscape contexts, and each included a 0.25-ha plantation, island treatment (trees planted in patches), and unplanted control. We analyzed four attributes of bird communities including frugivore abundance, nectarivore abundance, migrant insectivore richness, and compositional similarity of bird communities in restoration plots to bird communities in old-growth forests. All four bird community variables were greater in plantations and/or islands than in control treatments. Frugivore and nectarivore abundance decreased with increasing tree cover in the landscape surrounding restoration plots, whereas compositional similarity to old-growth forests was greatest in plantations embedded in landscapes with high tree cover. Migrant insectivore richness was unaffected by landscape tree cover. Our results agree with previous studies showing that increasing levels of investment in active restoration are positively related to bird richness and abundance, but differences in the effects of landscape tree cover on foraging guilds and community composition suggest that trade-offs between biodiversity conservation and bird-mediated ecosystem functioning may be important for prioritizing restoration sites.

## Introduction

Ecological restoration is the process of assisting the recovery of degraded ecosystems to their historic trajectories [1]. Interventions such as tree planting are effective for restoring biodiversity (*e.g.*, species diversity, abundances, or biomass) and ecosystem services (*e.g.*, nutrient cycling, soil stabilization, or climate regulation) in tropical terrestrial ecosystems [2]. Tropical restoration efforts help offset the impacts of ongoing deforestation [3] that threaten to exacerbate climate change and drive extinctions in the world's richest biological communities [4].

Restoration projects are spatially explicit, but rarely replicated across landscapes due to high implementation costs [5]. As such, our understanding of the importance of landscape context on the restoration of communities and their associated ecosystem functions and societal benefits is limited to a relatively small number of studies [6]–[10]. Nonetheless, funding for tropical forest restoration is increasingly available from national payment for ecosystem services programs and climate change mitigation initiatives such as Reducing Emissions from Deforestation and Forest Degradation (REDD+) [11]. Better understanding of landscape effects in tropical forest restoration will help inform sub-national prioritization criteria to effectively allocate limited funding and conform to environmental safeguards [12], [13].

We chose birds for this study because they are both beneficiaries and benefactors of forest restoration. Many tropical bird populations are in decline because of habitat loss [14], [15], but forest restoration aims to reverse this trend through gains in habitat [16]. Correspondingly, birds provide ecosystem functions that reduce biotic barriers to forest succession [17]–[19], including, seed dispersal [20]–[22], increased germination via gut passage [23], herbivorous arthropod control [24], and pollination [25], [26].

Early research on birds and tropical forest restoration showed that habitat structures such as isolated trees [27]–[30] and artificial perches [31]–[33] increased bird visitation and seed dispersal in degraded habitats, but isolated trees are not always available and seedling recruitment rarely increased below artificial perches [34]. Additional research demonstrated that mixed-species tree plantations both attracted seed dispersers and improved conditions for seed germination and seedling survival [35]–[40]. Seed dispersal trends were driven by small omnivores [41], [42], and the effects of tree planting varied by feeding guild [24], [26], [43] and habitat associations [44], [45]. Still, knowledge gaps remain [46]. In particular, few tropical forest restoration studies have been sufficiently replicated across the landscape to assess interactions between local restoration treatments and landscape context [44], [47].

The aim of this research was to learn how tropical forest restoration treatments interact with landscape tree cover to affect bird visitation and community composition in regenerating farmlands. We sought to identify differences in explanatory factors for avian foraging guilds versus birds associated with old-growth forest. We addressed these questions in a replicated restoration experiment in southern Costa Rica and found that both local treatments and landscape context were important predictors of bird community structure and composition.

## Materials and Methods

### Ethics Statement

Permission to conduct this research was granted by the Costa Rican Ministry of the Environment and Energy. Permission to work on private lands was granted by all landowners. This research did not require approval for animal care and use because it was an observational field study that did not involve the capture or handling of wild animals nor their maintenance in captivity.

### Study Area

We sampled bird communities in 13 restoration sites and two old-growth forests in southern Costa Rica (canton of Coto Brus). Restoration sites were located between the Las Cruces Biological Reserve (8° 47′ N, 82° 57′ W) and the town of Agua Buena (8° 44′ N, 82° 56′ W). Study sites were 1100–1400 m a.s.l., and the dominant natural ecosystem was premontane moist forest [48]. Most study sites were on the Fila Cruces, but one old-growth site was at similar elevation in the Talamanca Mountains. Precipitation across the study areas varies with topography but is ∼3.4 m y^−1^, with a mean annual temperature of 21°C at the Las Cruces Biological Station.

The study region was settled in the 1950s–80s by farmers from the Central Valley of Costa Rica and colonists from southern Italy [49]. Pioneers cleared the contiguous forest for coffee production, but many converted their coffee plantations to cattle pastures when coffee prices sunk in the 1990s [50]. The study area is currently a diverse mix of agricultural fields, coffee plantations, cattle pastures, small urban centers, and several types of forest elements including forest fragments, riparian forests, fencerows, and isolated trees. Forest elements comprise ∼35% of the study region [51].

### Experimental design

**Figure 1:**
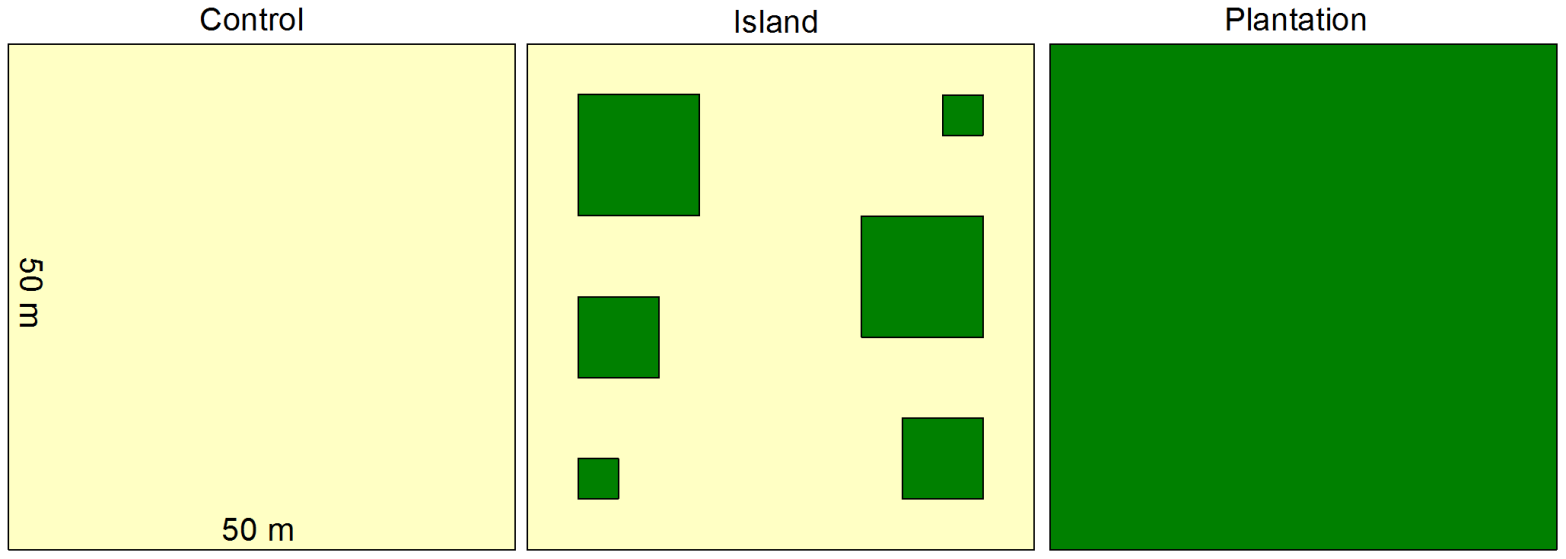
Local restoration treatments. Restoration treatments randomly applied to plots of heavily degraded pasture at 13 sites in 2004–2006 in southern Costa Rica. Green denotes areas planted with seedlings. Control plots were cleared of vegetation and allowed to regenerate naturally; islands were cleared and planted with 86 seedlings of four species in six patches (two each 4×4, 8×8, 12×12 m); plantations were cleared and planted in uniform rows throughout the plot (313 seedlings).

Restoration sites were established on degraded farmlands (mostly pastures) in 2004–2006. Each site included three 50×50 m plots (*N* = 39 plots), which were cleared of vegetation and randomly assigned to one of three treatments (Fig. 1). **Controls** were allowed to regenerate naturally; **islands** (*i.e.*, applied nucleation treatments) were planted with six tree seedling patches, or “islands”, of various sizes; and contiguous **plantations** were planted with rows of seedlings across the entire plot. The restoration treatments spanned a gradient of intervention intensity; tree seedling density (seedlings 0.25 ha^−1^) ranged from zero seedlings in controls, 86 in islands, to 313 in plantations. Seedlings planted in islands and plantations were a mix of two native timber species, *Terminalia amazonica* (J.F. Gmel.) Exell (Combretaceae) and *Vochysia guatemalensis* Donn. Sm. (Vochysiaceae), and two naturalized legumes, *Erythrina poeppigiana* Walp. Skeels and *Inga edulis* Mart. (Fabaceae). All restoration plots were cleared with machetes at ∼3-mo intervals for 2.5 y to allow planted seedlings to grow above existing grasses and forbs. Treatments had been in place for 3–7 y when bird counts took place at which time there were already large differences in vegetation structure and plant composition between treatments. See Cole *et al.* [9] for detailed descriptions of the restoration treatments and planted tree species selection.

Bird communities in restoration treatments were compared to a reference community sampled from two old-growth forests. The first old-growth forest site (Las Cruces: 8° 47′ N, 82° 57′ W) was located 1.2–8.9 km from restoration sites, and the second (La Amistad: 8° 56′ N, 82° 50′ W) was located 21.5–27.7 km from restoration sites. Each old-growth forest hosted six plots to sample the reference bird community (*N* = 12 plots).

### Bird surveys

Birds in restoration sites were surveyed seven times by a single observer, J.A.R., in Dec 2009 and Apr, Jul, and Nov 2010 and 2011. Each experimental plot at each site was actively searched in a random order for 20 min per observation, and all birds seen or heard within the plot were recorded. Old-growth forests were surveyed using point counts in May-Sep 2010. Each point count was conducted for 30 min by J. Figueroa-Sandí within a maximum radius of 50 m. Surveying methods differed because old-growth forests were initially sampled for a separate study [51]. We used these data to calculate an index of community similarity, but we did not directly compare bird communities in restoration and old-growth. All surveys were conducted from sunrise (∼5:30 AM) until 9:00 AM. Birds flying over sites without using them were excluded from analyses. Bird taxonomy follows the American Ornithologists' Union Checklist of the Birds of North America and its supplements [52].

We assigned bird species to three guilds (frugivores, migrant insectivores, nectarivores) based on published dietary descriptions [53]. We selected frugivores, migrant insectivores, and nectarivores based on their effects on ecosystem functions that benefit society (Table S1 in File S1). Frugivore abundance was a strong predictor of seed rain richness (*r* ^2^ = 0.95) and abundance (*r* ^2^ = 0.71) in the study area [54] (but see [55]), and migrant insectivore richness was the best community predictor of arthropod reduction in a coffee agroforestry system in southern Mexico (*r* ^2^ = 0.64) [56]. We assumed that nectarivore abundance would have greater power than species richness for predicting pollination because vertebrate pollination networks are characterized by low dependency [57]. Many species were omnivores, and some were classified in more than one guild. Because we were interested in guilds as they relate to ecosystem functioning, frugivores were limited to fruit-eating birds that also disperse viable seeds. Thus, seed predators (*i.e.*, Psittacids) and species that eat fruit but rarely defecate viable seeds in the study region were not included [41].

Observational data from restoration sites and trait data are publicly archived at https://merritt.cdlib.org/m/ucsc_lib_hollzahawi.

### Landscape tree cover

We used landscape tree cover as a measure of landscape context because it has performed well in previous studies [51], [58], [59]. Landscape tree cover was classified by manually digitizing aerial photographs from 2003 and 2005 with 2-m resolution [51]. Tree cover includes primary and secondary forest fragments of all sizes, single trees, early secondary growth, live fences, hedgerows, non-native timber and fruit tree plantations, and nonnative garden ornamentals. We calculated the percentage of landscape tree cover within 36 concentric rings around each restoration treatment (every 10 m from 10–200 m, every 50 m from 250–1000 m). This range of spatial scales includes scales that have been found to be relevant to bird communities in other studies [51], [60], [61].

### Data analysis

To compare community composition of birds in restoration plots and old-growth forests, we used a presence-absence matrix to calculate a Sørensen similarity index: QS_ij_ = 2C_ij_/S_i_+S_j_, where C is the number of species in common between sites *i* and *j* and *S* is the total number of species at a given site [62]. We selected a presence-absence similarity index rather than one based on abundance in order to account for differences in survey methods in old-growth forests and restoration sites. Migratory songbirds were excluded from the similarity analysis due to different sampling seasons for restoration sites and old-growth forests. No other seasonal trends were evident. We used a Mantel test of a similarity matrix and distance matrix to evaluate potential for spatial autocorrelation. After removing an outlying old-growth forest site (La Amistad) that was >21 km from all restoration sites, similarity values between sites were not explained by proximity (*r* = 0.19, *p* = 0.116, 9999 permutations).

We analyzed bird communities using linear mixed-effects models and maximum likelihood model selection [63], [64]. Response variables included frugivore and nectarivore abundance (detections 20-min observation^−1^), migrant insectivore richness (total number of species observed over two years of sampling), and compositional similarity of birds in restoration plots to birds in old-growth forest (QS).

General model structure was *y* = β_0_+β_1_ *x*_1_+/×β_2_ *x*_2_+s*_i_*+ε*_i_* where *y* is one of four bird community response variables, β_0_ is the y-intercept, β_1_ and β_2_ are vectors of fixed-effect coefficients for the three restoration treatments (*x*_1_) and landscape tree cover (*x*_2_), *s_i_* is the random effect for the *i*th site, and ε*_i_* is the error term. Responses were modeled using log-link for Poisson-distributed responses (frugivore and nectarivore abundance and migrant insectivore richness) and identity-link for normally-distributed responses (compositional similarity to old-growth forest). Spatial and temporal non-independence of treatments within sites were modeled as nested random effects.

For each response variable, we tested models that included fixed effects for: (1) restoration treatment only, (2) restoration treatment + tree cover, and (3) restoration treatment × tree cover. For model types that included tree cover, we compared 36 individual models with tree cover calculated within different-sized concentric rings (10–1000 m radius) around each experimental plot at each site. To avoid collinearity, we used a separate model for each buffer scale. We used Akaike Information Criterion (AIC) scores and weights corrected for small sample sizes (AIC_c_) to select the best model from each set. We report the model with the lowest AIC_c_ score as well as the range of buffer scales that resulted in models with ΔAIC_c_<2. Effects of local restoration treatments on avian communities were also analyzed using non-parametric Kruskal-Wallis and Wilcoxon rank sum tests with Bonferroni corrections. For the frugivore abundance analysis, we excluded one site from the model selection procedure because it had a disproportionate influence for models with small tree cover radii. Significance of individual fixed factors was assessed by removing one factor from the model and comparing AIC_c_ scores. To assess whether patterns observed at the community level made good predictions at the individual species level, we inspected plots of the most abundant species in each group across sites and tree cover gradients. Analyses were conducted in R 2.15.0 [65] using the lme4 package [66].

Our analytical approach addresses several common criticisms of bird community studies in conservation biology. First, species richness metrics and forest dependency indices are superficial measures of biodiversity and its responses to change [67], [68]. We avoided this issue by using similarity to old-growth forest as a response variable rather than abundance or richness of forest-dependent birds. Second, recent studies have highlighted heterogeneous bird detectability in different habitats, which may confound cross-habitat comparisons [69]. We addressed this problem by intensively surveying small areas, where detection probability was likely high and assumptions of occupancy models could not be met. Implications of this decision are considered in the discussion. Third, many studies compare birds across small spatial scales and are biased by spatial autocorrelation [70]. Sites in this study were sufficiently spaced (>700 m separation), but treatments within sites were separated by only ∼10–200 m. This spatial arrangement was ideal for our study because we were interested in relative habitat visitation by birds presented with a choice of three restoration treatments at each site. Spatial non-independence was therefore modeled as a nested random effect. Finally, studies evaluating vertebrate responses to small habitat manipulations have unique challenges and should be explicit about the inferences that can be made [71]. We do not assume that any birds complete their life cycle within the restoration sites that we studied or that these interventions have restored bird communities per se. Rather, we infer that bird visitation denotes that restored habitat supports one or more aspects of a bird's ecology.

## Results

We observed 3852 bird visitations to restoration sites representing 125 species from 29 families (Figures S1–S2 in File S1). Avian guilds and compositional similarity to old-growth forest differed among local restoration treatments (all Χ^2^≥14; *p*<0.001). Plantations had significantly greater frugivore and nectarivore abundance, migrant insectivore richness, and compositional similarity to old-growth forest birds than controls (all *p*<0.01) and islands were intermediate (Table S2 in File S1).

**Figure 2:**
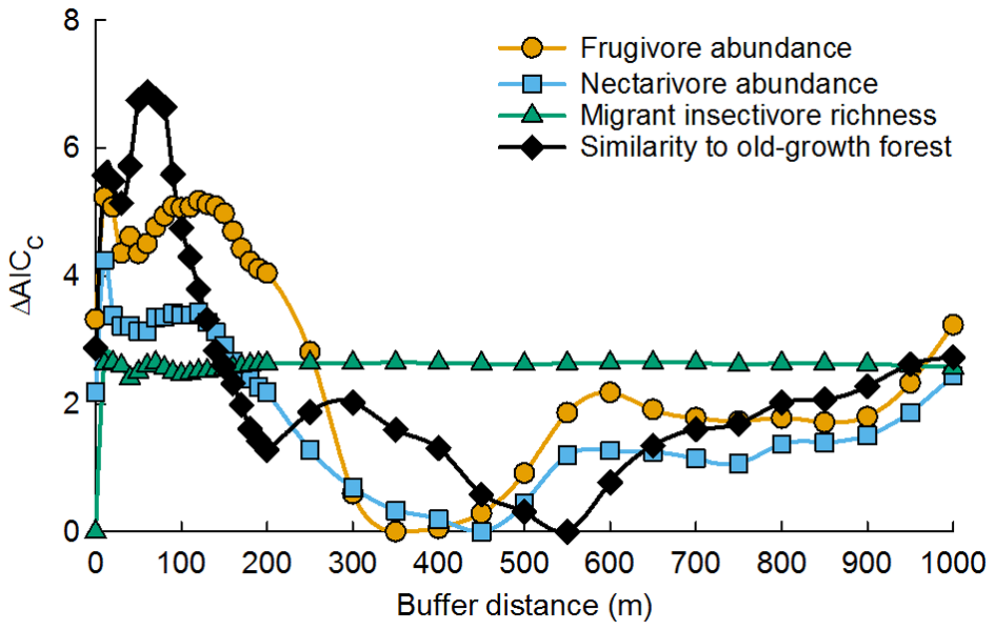
Landscape tree cover model comparison. Model comparison for community similarity to old-growth forest (diamonds) and foraging guilds (frugivores  =  circles; nectarivores  =  squares; migrant insectivores  =  triangles) predicted by tree cover at varying buffer distances around restoration sites. AIC_c_ represents an Akaike Information Criterion score corrected for small sample sizes. ΔAIC_c_ represents the difference in AIC_c_ scores between a given model and the model with the lowest AIC_c_.

Supported models included local restoration treatments and landscape tree cover for frugivore abundance (range of tree cover buffer scales with ΔAIC_c_<2: 300–900 m; best-fit buffer scale: 350 m), nectarivore abundance (range: 250–950 m; best: 450 m), and compositional similarity to old-growth forest (range: 170–250, 350–750 m; best: 550 m) (Fig. 2, Table S3–4 in File S1). Migrant insectivore richness was only predicted by local restoration treatment (Table 1).

**Table 1:** Model estimates for bird community attributes in tropical forest restoration.

Response variable	Model fit adj. *r* ^2^/*P*	Parameter	Level	Estimate	s.e.m.
similarity to old-growth forest	0.83/<0.001	intercept	-	0.139	0.034
		treatment	island	0.052	0.036
			plantation	−0.002	0.037
		tree cover	550 m	−0.052	0.071
		interaction	island × tree cover	0.023	0.077
			plantation × tree cover	0.268	0.081
frugivore abundance	0.59/<0.001	intercept	-	1.544	0.302
		treatment	island	0.734	0.117
			plantation	1.243	0.111
		tree cover	350 m	−1.255	0.573
migrant insectivore richness	0.54/<0.001	intercept	-	0.523	0.215
		treatment	island	1.068	0.248
			plantation	1.291	0.241
nectarivore abundance	0.36/<0.001	intercept	-	0.713	0.263
		treatment	island	0.292	0.131
			plantation	1.195	0.126
		tree cover	450 m	−1.089	0.516

**Figure 3:**
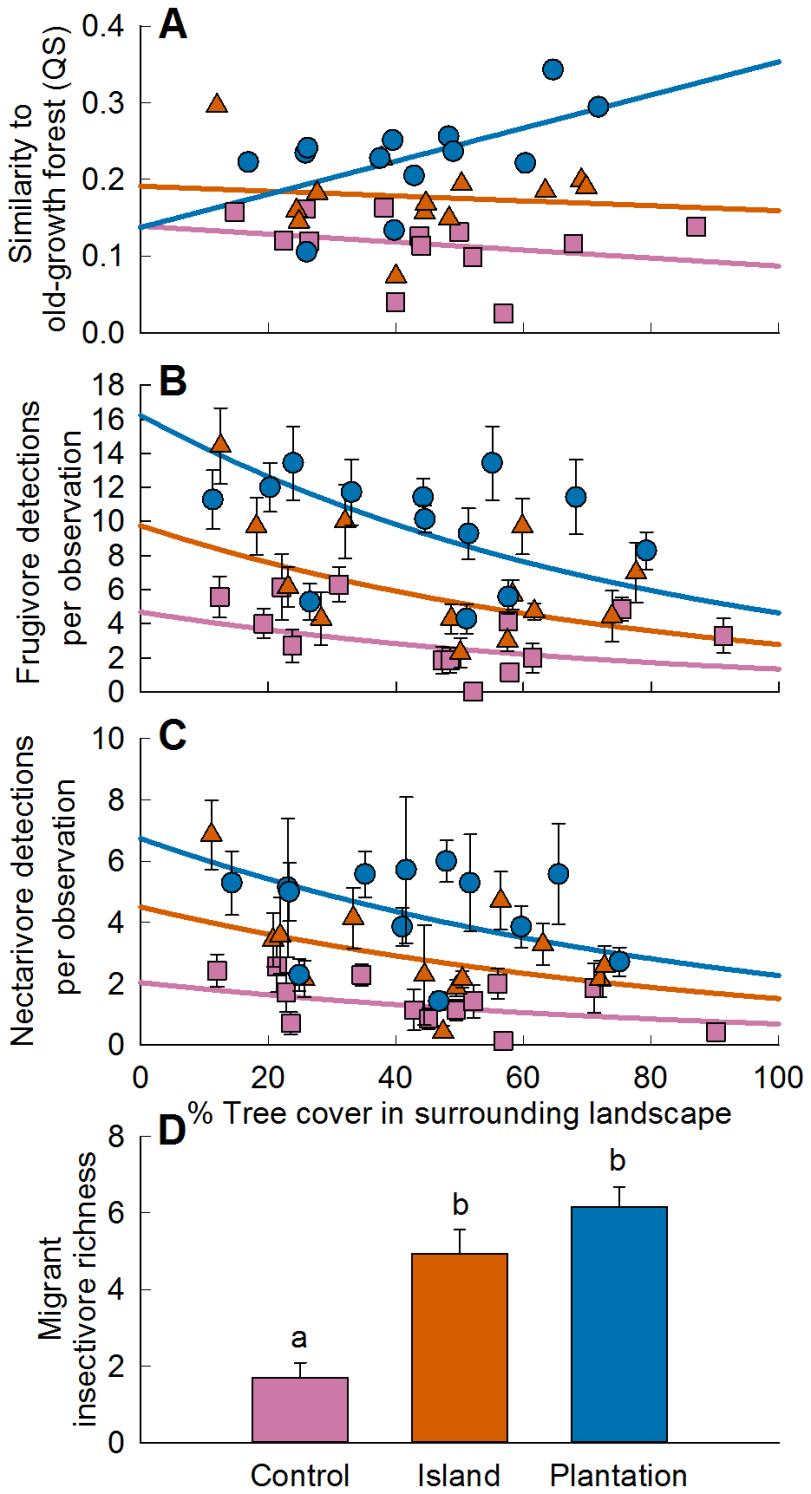
Compositional and functional attributes of bird communities predicted by restoration treatments and landscape tree cover. Error bars represent ±1 s.e.m. (A–C) Controls are represented by squares; islands by triangles, and plantations by circles. (A) Sørensen similarity of bird communities in restoration sites to bird communities in reference forest (550 m tree cover buffer); (B) frugivore abundance per observation (7 observations per point; 350 m buffer); (C) nectarivore abundance per observation (450 m buffer); (D) migrant insectivore richness (equal sampling intensity). Significance calculated using pairwise Wilcoxon rank sum tests with Bonferroni corrections (all P<0.004).

The best-fit model for predicting compositional similarity to old-growth forest included an interaction between local restoration treatment and landscape tree cover (Fig. 3A). Bird communities in plantations more closely resembled bird communities in old-growth forest plots when there was greater landscape tree cover within a radius of 550 m (adj. *r* ^2^ = 0.83, *p*<0.001; Table 1, Tables S3-S4 in File S1). An outlying island plot with low tree cover and relatively high compositional similarity appeared to drive results for island plots (Fig. 3A). When we analyzed the data with this outlier removed, the coefficient for tree cover effect on island plots was greater (0.122±0.079 SE) than with the outlier included (0.023±0.077), however this difference had little impact over the range of possible tree cover values. Results were not substantially different when we ran the analysis excluding two sites that were immediately adjacent to old-growth forest patches.

Frugivore and nectarivore relative abundance declined as landscape tree cover increased within radii of 350 m and 450 m, respectively (Fig. 3B–C), but local treatment × landscape tree cover interactions were not significant. The effect of landscape tree cover was not apparent in the trends of individual species (Table S5 in File S1). Of the ten most abundant species in each analysis group, eight frugivores, seven nectarivores, nine migratory insectivores, and eight reference forest species were detected more frequently in plantations than controls (Table S5 in File S1). Five of the most frequently detected species in old-growth forests increased in plantations relative to controls as tree cover increased within a radius of 550 m. No individual species had a negative relationship with landscape tree cover.

## Discussion

Our results show that local restoration and landscape context interacted to affect bird community composition, but not foraging guilds. Compared to less intensive restoration practices, tree plantations had: (1) greater abundance of frugivores and nectarivores, (2) greater migratory insectivore species richness; and (3) greater compositional similarity to old-growth forest. Similarity to old-growth bird communities increased with greater tree cover in the surrounding landscape, but only in plantations. In contrast, frugivore and nectarivore abundance and migrant insectivore richness were greatest in tree plantations regardless of landscape tree cover, and frugivore and nectarivore abundance were lower in landscapes with high tree cover.

Spatial scaling of landscape effects was similar (best models: 0.35–0.55 km radius) for old-growth similarity, frugivores, and nectarivores, but the direction of these effects was surprisingly different. Variance in local × landscape interactions for old-growth similarity and foraging guilds may be best explained by dispersal limitation and niche complementarity. Guilds are delineated on the basis of species traits, such as diet [19], but species identity is central to measures of community composition [72] and associated conservation values. High similarity to old-growth bird communities in plantations embedded within well-forested landscapes are contingent upon birds dispersing from pre-existing forest into restoration sites [10]. Strong evidence shows that some forest birds are unable to cross even small distances through unusable habitat [73], and that the most dispersal-limited species are typically also the most prone to extinction in fragmented landscapes [74]. Fragmentation studies have often highlighted that terrestrial insectivores are among the most extinction-prone birds [75]. Likewise, we found that the most frequently detected species in old-growth forest was a terrestrial insectivore, *Formicarius analis*, which was only recorded in two restoration sites, one of which was adjacent to an old-growth forest fragment. Intensive local restoration efforts may thus provision suitable habitat for forest-dependent species, but their colonization depends upon matrix permeability and the composition of regional species pools [76], [77].

In contrast to old-growth forest birds, frugivores, nectarivores, and migrant insectivores were more abundant or speciose in tree plantations than in less-intensive restoration treatments regardless of landscape context. This observation could result from niche complementarity – the tendency of species similar on one niche axis to differ along another. In our study area, frugivores, nectarivores, and insectivores that are otherwise similar (sometimes congeneric) are separated by habitat affinity into partially overlapping agricultural and forest communities [51]. The result is a portfolio effect, where reductions in forest-affiliated frugivores, for example, are balanced by increased abundance of agriculture-affiliated frugivores, potentially maintaining a constant level of bird-mediated ecosystem functions despite high species turnover [78], [79]. However, guild classifications simplify functional heterogeneity. For example, frugivore trends in this study were driven by small omnivores, whereas wide-gaped species that could disperse large seeds were virtually absent. In this context, intensive local restoration in sites with low landscape tree cover may attract a subset of agriculture-affiliated birds already present in the surrounding matrix by provisioning food resources, favorable microclimate, or cover from diurnal predators [80], [81].

Subtle community-wide increases in frugivore and nectarivore detections in sites with lower tree cover may be explained by the marginal value theorem of optimal foraging [82]. If lower tree cover in the surrounding landscape indicates greater travel distances between patches, then birds may spend more time and potentially be detected more frequently exploiting food resources in restoration sites with little tree cover in the surrounding landscape. This observation suggests that smaller forest elements become more valuable (to a subset of the regional bird species pool) when they make up larger proportions of local forest cover. A lack of landscape tree cover effect on migrants compared to other groups could be due to territorial exclusion if individuals are commonly relegated to low quality patches [83] or from a preference for early-successional habitats [84]. Alternately, hierarchical landscape selection by migrants could occur at a spatial scale larger than the maximum buffer of 1 km used here [85].

Differences in landscape effects on old-growth forest species and foraging guilds support the hypothesis that restoration does not necessarily optimize biodiversity conservation and ecosystem functioning simultaneously [86]. A growing body of literature is establishing connections between biodiversity and ecosystem functioning (BEF) [87]–[89] (but see [90]), but trade-offs between ecosystem services like carbon sequestration and conservation-relevant biodiversity outcomes are evident for ecological restoration at national and global scales [91], [92]. Terminology is a primary hang-up. *Biodiversity* in the BEF conversation is taken to include taxonomic, phylogenetic, genetic, functional, spatial, temporal, interaction, and landscape diversity [89], but conservation priorities are typically designated using other biodiversity concepts, such as the richness or abundance of threatened, endemic, and forest-affiliated species, population diversity, and community composition [68], [93], [94]. Our data suggest that this divide between basic and applied biodiversity-ecosystem function science may extend to bird communities if bird-mediated functions are redundant across species or do not align with species-specific conservation priorities.

The local × landscape interaction that we detected for predicting old-growth species composition has implications for allocating restoration funds at sub-national scales. Large-scale tropical forest restoration is on the rise, due in large part to increased funding from REDD+ and various payments for ecosystem services programs [11], [95], [96]. Our data suggest that new restoration projects in areas with high forest cover will likely promote greater colonization by species representative of reference communities – and thus safeguard biodiversity conservation – than similar projects in habitat-poor landscapes [97]. Also, more intensive local interventions are likely to benefit birds over a 5–7 yr period more than less intensive or passive restoration techniques [26], [41], [98]. Given time, we expect that effects of local restoration treatments will converge as low-intensity control plots increasingly resemble closed-canopy forest [43], [99], but landscape effects are likely to endure.

An alternative explanation for differences in observed bird visitations between local restoration treatments could be that detectability varied among treatments [69], [100]. We were unable to evaluate detection probabilities, but we expect that a habitat-specific bias would favor increased bird detections in open control plots relative to closed-canopy plantations. Such a bias would strengthen our conclusion that relative abundance is greater in plantations, however, this result does not denote a successful restoration of the bird community per se in any particular treatment [101]. Many habitat manipulation studies, including this one, are too small to reliably detect differences in population or demographic variables needed to infer community restoration, but they still contain useful information [71].

We have demonstrated experimentally that intensive local restoration of degraded pastures promotes three functionally-relevant foraging guilds regardless of surrounding tree cover. Also, intensive restoration coupled with high amounts of surrounding tree cover increases habitat for species affiliated with old-growth forest within a few years of the intervention. We note that while ecological restoration holds great hope for slowing or reversing the tide of biodiversity loss [16], restoration cannot be considered a substitute for the preservation of existing forest [102].

## Supporting Information

File S1 Table S1. Correlates of bird-mediated seed dispersal, arthropod control, and pollination in tropical ecosystems. Table S2. Effects of local restoration treatments on bird community attributes. Table S3. Maximum likelihood model selection for bird community attributes. Table S4. Maximum likelihood tests for significance of fixed factors explaining bird community attributes. Table S5. Detection trends for common species in each group. Figure S1. Weights and family composition of bird species detected in restoration sites. Figure S2. Detections and species composition of large frugivores (weight >100 g) in restoration sites.
(DOCX)
