# Supplementary material for: Landscape Context Mediates Avian Habitat Choice in Tropical Forest Restoration
Source: PLoS One. 2014 Mar 4;9(3):e90573. doi: 10.1371/journal.pone.0090573 (PMC3942431; doi:10.1371/journal.pone.0090573)
Supplement: File S1 — Table S1. Correlates of bird-mediated seed dispersal, arthropod control, and pollination in tropical ecosystems. Table S2. Effects of local restoration treatments on bird community attributes. Table S3. Maximum likelihood model selection for bird community attributes. Table S4. Maximum likelihood tests for significance of fixed factors explaining bird community attributes. Table S5. Detection trends for common species in each group. Figure S1. Weights and family composition of bird species detected in restoration sites. Figure S2. Detections and species composition of large frugivores (weight >100 g) in restoration sites. (DOCX) [file pone.0090573.s001.docx]

**Supporting Information**

**Table S1** Correlates of bird-mediated seed dispersal, arthropod control, and pollination in tropical ecosystems.

| Bird community attribute (x) | Ecosystem function (y) | Relationship (*R*^2^) |
| --- | --- | --- |
| Frugivore abundance | Dispersed seed richness | ŷ = 0.876x – 5.070 (0.95)^*^ |
|  | Dispersed seed abundance | ŷ = 0.202x – 2.408 (0.71)^*^ |
| Migrant insectivore richness | % Reduction in total arthropod abundance | ŷ = 10.029x – 40.785 (0.64)^†^ |
|  | % Reduction in large arthropod abundance | ŷ = 8.7336x – 8.3759 (0.68)^†^ |
| Nectarivore abundance | Quantity and quality of flower pollination | *na*^‡^ |

^*^ Pejchar et al. (2008) *Biol Conserv* **141**, 536–544.

^†^ van Bael et al. (2008) *Ecology* **89**, 928–934.

^‡^No study has yet related attributes of avian nectarivore communities to pollination functions. We use nectarivore abundance rather than species richness because vertebrate pollination networks are characterized by low dependency.

**Table S2** Local restoration effects on avian community attributes. Significant differences (α = 0.05) from Wilcoxon rank sum tests with Bonferroni corrections are denoted by ^AB^.

|  | Control | Island | Plantation | Χ^2^ | *p* |
| --- | --- | --- | --- | --- | --- |
| Frugivores (detections/observation) | 3.3 ± 0.4^A^ | 6.8 ± 0.5^B^ | 9.7 ± 0.5^B^ | 16.4 | <0.001 |
| Nectarivores (detections/observation) | 1.4 ± 0.2^A^ | 3.0 ± 0.3^B^ | 4.4 ± 0.4^B^ | 19.0 | <0.001 |
| Migrant insectivores (observed species richness) | 1.7 ± 0.4^A^ | 4.9 ± 0.6^B^ | 6.2 ± 0.5^B^ | 19.6 | <0.001 |
| Similarity to old-growth (QS) | 0.12 ± 0.01^A^ | 0.18 ± 0.01^A^ | 0.23 ± 0.02^B^ | 18.8 | <0.001 |

**Table S3** Maximum likelihood model selection for bird community attributes.

| Response variable | Model (fixed effects) | K | ΔAIC_c_ | w_i_ |
| --- | --- | --- | --- | --- |
| Similarity to reference forest | int + treatment | 5 | 2.87 | 0.24 |
|  | int + treatment + tree cover | 6 | 4.88 | 0.87 |
|  | **int + treatment × tree cover** | **8** | **0.00** | **1.00** |
| Frugivore abundance | int + treatment | 5 | 3.31 | 0.19 |
|  | **int + treatment + tree cover** | **6** | **0.00** | **1.00** |
|  | int + treatment × tree cover | 8 | 1.41 | 0.49 |
| Migrant insectivore richness | **int + treatment** | **5** | **0.00** | **1.00** |
|  | int + treatment + tree cover | 6 | 2.62 | 0.27 |
|  | int + treatment × tree cover | 8 | 2.15 | 0.34 |
| Nectarivore abundance | int + treatment | 5 | 2.19 | 0.34 |
|  | **int + treatment + tree cover** | **6** | **0.00** | **1.00** |
|  | int + treatment × tree cover | 8 | 0.62 | 0.73 |

**Table S4** Maximum likelihood tests for significance of fixed factors explaining bird community attributes

| Response variable | Hypothesis | ΔAIC | Χ^2^ | *p* |
| --- | --- | --- | --- | --- |
| Similarity to reference forest | β(treatment) = 0 | -32.0 | 40.0 | <0.001 |
|  | β(tree cover) = 0 | -5.8 | 11.8 | 0.008 |
|  | β(interaction) = 0 | -7.8 | 11.8 | 0.003 |
| Frugivore abundance | β(treatment) = 0 | -114.0 | 118.0 | <0.001 |
|  | β(tree cover) = 0 | -2.7 | 4.7 | 0.029 |
| Migrant insectivore richness | β(treatment) = 0 | -33.0 | 37.0 | <0.001 |
| Nectarivore abundance | β(treatment) = 0 | -78.3 | 82.3 | <0.001 |
|  | β(tree cover) = 0 | -2.3 | 4.3 | 0.039 |

**Table S5** Individual trends for the ten most abundant bird species in each group (frugivores, nectarivores, migrant insectivores, and species found in old-growth forest). Taxonomy follows the American Ornithologists’ Union (2007) and its supplements. Species are ordered by group and by the number of detections or by the number of occurrences in old-growth forest plots (Old-growth forest species).

| Group | Family | Species | Detections (N) | % Detections in group (%) | Sites with greater observations in plantations than controls / sites where species occurred | Trend in abundance with increasing tree cover |
| --- | --- | --- | --- | --- | --- | --- |
| Frugivore | Thraupidae | *Ramphocelus costaricensis* | 273 | 15.0 | 7/13 | none |
|  | Parulidae | *Basileuterus rufifrons* | 134 | 7.4 | 10/11 | none |
|  | Incertae Sedis | *Saltator maximus* | 124 | 6.8 | 8/13 | none |
|  | Turdidae | *Catharus ustulatus* | 120 | 6.6 | 11/13 | none |
|  | Turdidae | *Catharus aurantiirostris* | 108 | 6.0 | 8/13 | none |
|  | Thraupidae | *Thraupis episcopus* | 96 | 5.3 | 6/12 | none |
|  | Parulidae | *Oreothlypis peregrina* | 95 | 5.2 | 13/13 | none |
|  | Incertae Sedis | *Saltator striatipectus* | 89 | 4.9 | 3/12 | none |
|  | Tyrannidae | *Zimmerius vilissimus* | 75 | 4.1 | 7/12 | none |
|  | Thraupidae | *Tangara larvata* | 72 | 4.0 | 11/12 | none |
| Nectarivore | Trochilidae | *Amazilia tzacatl* | 211 | 26.0 | 12/13 | none |
|  | Incertae Sedis | *Saltator maximus* | 122 | 15.0 | 8/13 | none |
|  | Thraupidae | *Thraupis episcopus* | 96 | 11.8 | 6/13 | none |
|  | Parulidae | *Oreothlypis peregrina* | 92 | 11.3 | 13/13 | none |
|  | Emberizidae | *Chlorospingus ophthalmicus* | 65 | 8.0 | 3/4 | none |
|  | Incertae Sedis | *Coereba flaveola* | 46 | 5.7 | 7/11 | none |
|  | Trochilidae | *Phaethornis guy* | 39 | 4.8 | 9/11 | none |
|  | Picidae | *Melanerpes rubricapillus* | 36 | 4.4 | 9/9 | slight decrease |
|  | Trochilidae | *Amazilia Edward* | 30 | 3.7 | 3/9 | none |
|  | Icteridae | *Psarocolius decumanus* | 20 | 2.5 | 1/3 | none |
| Migrant insectivore | Turdidae | *Catharus ustulatus* | 120 | 25.2 | 11/13 | none |
|  | Parulidae | *Setophaga pensylvanica* | 99 | 20.8 | 12/12 | none |
|  | Parulidae | *Oreothlypis peregrina* | 92 | 19.3 | 13/13 | slight decrease |
|  | Parulidae | *Geothlypis philadelphia* | 67 | 14.1 | 7/11 | none |
|  | Parulidae | *Cardellina pusilla* | 29 | 6.1 | 7/12 | none |
|  | Parulidae | *Setophaga fusca* | 19 | 4.0 | 7/9 | none |
|  | Icteridae | *Icterus galbula* | 13 | 2.7 | 5/6 | none |
|  | Parulidae | *Mniotilta varia* | 11 | 2.3 | 4/6 | none |
|  | Parulidae | *Setophaga petechia* | 8 | 1.7 | 3/5 | none |
|  | Cardinalidae | *Piranga rubra* | 7 | 1.5 | 2/5 | slight increase |
| Old-growth forest species | Formicariidae | *Formicarius analis* | 4 (12) | 0.2 | 2/2 | increase |
|  | Vireonidae | *Hylophilus decurtatus* | 7 (12) | 0.3 | 1/4 | none |
|  | Troglodytidae | *Henicorhina leucosticta* | 34 (12) | 1.5 | 5/6 | increase |
|  | Emberizidae | *Arremon aurantiirostris* | 1 (11) | 0.0 | 0/1 | none |
|  | Emberizidae | *Chlorospingus ophthalmicus* | 65 (10) | 2.8 | 3/4 | increase |
|  | Tyrannidae | *Zimmerius vilissimus* | 73 (10) | 3.2 | 7/12 | none |
|  | Tyrannidae | *Lophotriccus pileatus* | 30 (10) | 1.3 | 10/10 | increase |
|  | Pipridae | *Corapipo altera* | 3 (10) | 0.1 | 2/2 | increase |
|  | Turdidae | *Turdus assimilis* | 9 (10) | 0.4 | 4/5 | none |
|  | Momotidae | *Momotus momota* | 15 (9) | 0.6 | 5/9 | none |


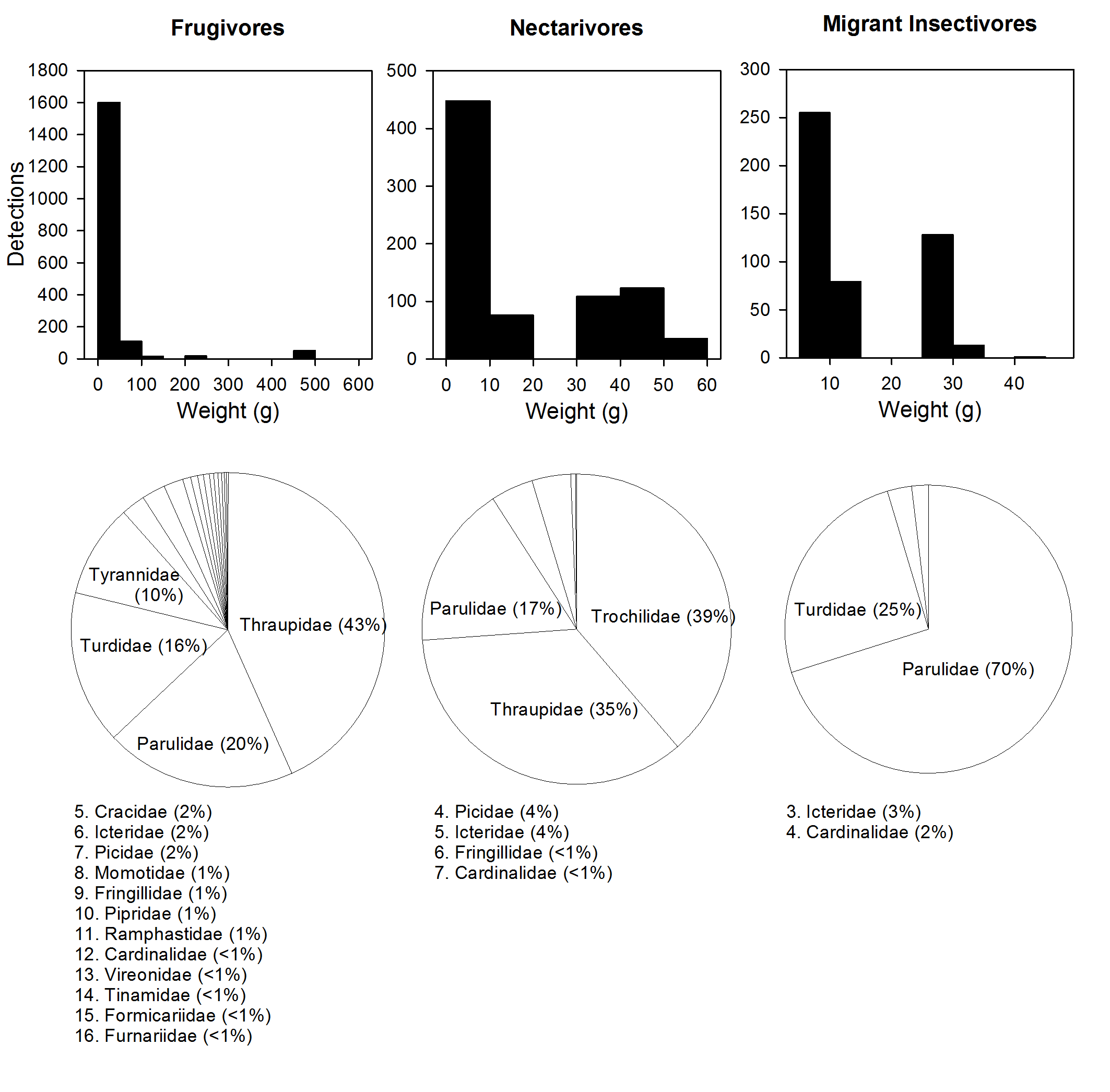


**Figure S1** Weights and family composition of bird species detected in restoration sites in southern Costa Rica. Weights are from Stiles and Skutch (1989). One large frugivore, *Psarocolius decumanus* (20 detections), is not shown.


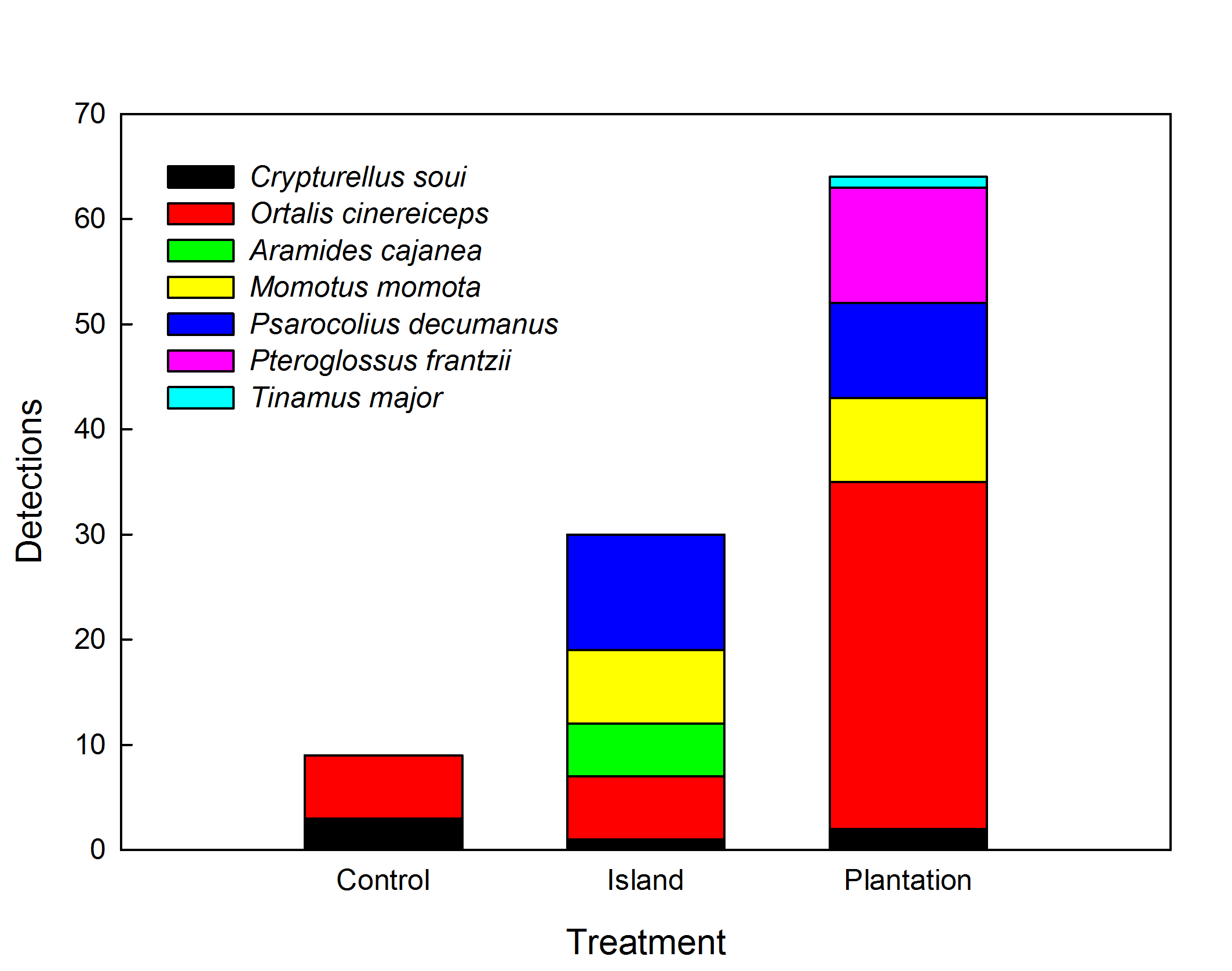


**Figure S2** Detections and composition of large frugivores (>100 g) in restoration sites in southern Costa Rica.
